# Breast adenocarcinoma liver metastases, in contrast to colorectal cancer liver metastases, display a non-angiogenic growth pattern that preserves the stroma and lacks hypoxia

**DOI:** 10.1038/sj.bjc.6601727

**Published:** 2004-03-09

**Authors:** F Stessels, G Van den Eynden, I Van der Auwera, R Salgado, E Van den Heuvel, A L Harris, D G Jackson, C G Colpaert, E A Van Marck, L Y Dirix, P B Vermeulen

**Affiliations:** 1 Translational Cancer Research Group Antwerp, Department of Pathology, University Hospital, University of Antwerp, Edegem, Belgium; 2 Translational Cancer Research Group Antwerp, Departments of Pathology and Oncology, General Hospital Sint-Augustinus, Wilrijk, Belgium; 3 Molecular Oncology Laboratory, Weatherall Institute of Molecular Medicine, John Radcliffe Hospital, Headington, Oxford, UK; 4 MRC Human Immunology Unit, Weatherall Institute of Molecular Medicine, John Radcliffe Hospital, Headington, Oxford, UK

**Keywords:** growth pattern, liver metastasis, angiogenesis, hypoxia, breast cancer, colorectal cancer

## Abstract

Although angiogenesis is a prerequisite for the growth of most human solid tumours, alternative mechanisms of vascularisation can be adopted. We have previously described a non-angiogenic growth pattern in liver metastases of colorectal adenocarcinomas (CRC) in which tumour cells replace hepatocytes at the tumour–liver interface, preserving the liver architecture and co-opting the sinusoidal blood vessels. The aim of this study was to determine whether this replacement pattern occurs during liver metastasis of breast adenocarcinomas (BC) and whether the lack of an angiogenic switch in such metastases is due to the absence of hypoxia and subsequent vascular fibrinogen leakage. The growth pattern of 45 BC liver metastases and 28 CRC liver metastases (73 consecutive patients) was assessed on haematoxylin- and eosin-stained tissue sections. The majority of the BC liver metastases had a replacement growth pattern (96%), in contrast to only 32% of the CRC metastases (*P*<0.0001). The median carbonic anhydrase 9 (CA9) expression (M75 antibody), as a marker of hypoxia, (intensity × % of stained tumour cells) was 0 in the BC metastases and 53 in the CRC metastases (*P*<0.0001). There was CA9 expression at the tumour–liver interface in only 16% of the BC liver metastases *vs* 54% of the CRC metastases (*P*=0.002). There was fibrin (T2G1 antibody) at the tumour-liver interface in only 21% of the BC metastases *vs* 56% of the CRC metastases (*P*=0.04). The median macrophage count (Chalkley morphometry; KP-1 anti-CD68 antibody) at the interface was 4.3 and 7.5, respectively (*P*<0.0001). Carbonic anhydrase 9 score and macrophage count were positively correlated (*r*=0.42; *P*=0.002) in all metastases. Glandular differentiation was less in the BC liver metastases: 80% had less than 10% gland formation *vs* only 7% of the CRC metastases (*P*<0.0001). The liver is a densely vascularised organ and can host metastases that exploit this environment by replacing the hepatocytes and co-opting the vasculature. Our findings confirm that a non-angiogenic pattern of liver metastasis indeed occurs in BC, that this pattern of replacement growth is even more prevalent than in CRC, and that the process induces neither hypoxia nor vascular leakage.

Blood vessels have comparable functions during organogenesis and tumour growth. These include the maintenance of blood flow necessary for delivery of oxygen and nutrients, as well as bidirectional paracrine interactions between endothelium and epithelium that influence proliferation, migration and differentiation of both cell populations (Cleaver and Melton, 2003). In most malignant human tumours, the vasculature results from angiogenesis, as evidenced by the formation of a desmoplastic stroma containing small immature blood vessels clustered in vascular ‘hot spots’, and by a relatively high fraction of proliferating endothelial cells. Vascularisation in human solid tumours is measured in these hot spots, which arise through angiogenesis and ongoing vessel remodelling, and this measure has a widely confirmed prognostic value in breast cancer (Vermeulen *et al*, 2002). Blood vessel growth is an invasive process that destroys the surrounding tissue architecture (Masson *et al*, 1998). Angiogenesis has traditionally been regarded as the sole mechanism for a malignant tumour to obtain a functional vasculature. The dormancy concept consolidated this view: when the number of capillaries in the tumour tissue in an animal model decreased, tumour cell proliferation was not affected but the apoptotic fraction increased, leading to ‘dormant’ tumours (Holmgren *et al*, 1995).

In 1997, however, simple morphological observations in human primary non-small-cell lung carcinomas led to the introduction of a new concept of tumour vascularisation: ‘co-option’ of blood vessels of the surrounding normal parenchyma appeared to be an efficient alternative for angiogenesis (Pezzella *et al*, 1997). Subsequently, experiments in a rat glioma model suggested that even angiogenic tumours initially co-opt normal blood vessels after which a host defence response, governed by angiopoietin-2 expression on the co-opted endothelial cells, causes blood vessel regression with concomitant hypoxia and vascular endothelial growth factor (VEGF)-mediated angiogenesis (Holash *et al*, 1999).

The liver has a comparably dense vasculature as the lungs and both organs frequently host metastases of carcinomas. The hypothesis of our first study therefore was that metastases that would be capable of preserving the stromal structure of the liver might not become hypoxic and thus would not be dependent upon angiogenesis for survival (Vermeulen *et al*, 2001). One of three growth patterns of colorectal adenocarcinomas (CRC) metastases in the liver, the replacement pattern, in which hepatocytes were merely replaced by tumour cells, forming muralia that were in continuity with the liver cell, was indeed characterised by co-option of sinusoidal blood vessels at the tumour–liver interface, by a lack of inflammation and desmoplastic stroma, and by a low fraction of proliferating endothelial cells. Sinusoidal blood vessels do not express CD34 and the co-opted vessel endothelium only started to express this endothelial cell marker when they were engulfed by a few rows of CRC cells. Tumour cell plasticity is also exemplified by recently described alternative mechanisms of intravasation (tumour cells express an endothelial-like phenotype during vasculogenic mimicry (Hendrix *et al*, 2003) and are part of the vessel wall) and of extravasation (intravascular growth of tumour cell nests results in metastasis without the need for extravasation; Alpaugh *et al*, 2002).

We have shown that cutaneous breast adenocarcinoma (BC) deposits are a heterogeneous group with different degrees of hypoxia-driven angiogenesis reflected in an infiltrative and an expansive growth pattern (Colpaert *et al*, 2003a). Using the marker carbonic anhydrase 9 (CA9) (Wykoff *et al*, 2000), we found evidence of hypoxia in only 17% of the cutaneous deposits with an infiltrative growth compared with 71% of the tumours with an expansive, nodule-forming growth (*P*=0.02). The infiltrative growth pattern was further characterised by a relatively low endothelial cell proliferation index (5 *vs* 18%; *P*=0.004), and fibrin deposition in a smaller fraction of the metastases (44 *vs* 100%; *P*=0.07). The cutaneous deposits with an infiltrative growth respected the dermal architecture and co-opted pre-existing collagen bundles, blood vessels and skin adnexa.

The aim of this study was to analyse the architecture of liver metastases of BC and CRC with emphasis on the presence or absence of angiogenesis and hypoxia. Radiological imaging studies have revealed subgroups of liver metastases with different contrast enhancement and delineation, supporting the hypothesis of morphological heterogeneity (Yamaguchi *et al*, 2002).

## MATERIALS AND METHODS

Tissue specimens of formalin-fixed, paraffin-embedded human liver metastases from 49 patients with BC and from 28 patients with CRC were retrieved from the files of the pathology departments of the General Hospital Sint-Augustinus and of the University Hospital of Antwerp. Twenty-eight BC metastases were necropsy-derived. The other metastases were obtained after elective surgery or needle biopsy. One tissue block, containing a representative fraction of the tumour–liver parenchyma interface, was used per patient.

Sections 5 μm in size were cut. A standard haematoxylin and eosin stain was carried out to evaluate the growth pattern based on the morphology of the tumour–liver parenchyma interface, as described before (Vermeulen *et al*, 2001).

In the ‘desmoplastic’ growth pattern, the metastases were separated from the surrounding liver parenchyma by a rim of desmoplastic stroma in which a dense mononuclear infiltrate and numerous capillaries were present. Often tumour cell nests were infiltrating the stroma. The tissue architecture of the liver was not conserved within the metastases.

In the ‘pushing’ growth pattern, liver plates were pushed aside and ran in parallel with the circumference of the metastases at the tumour–liver parenchyma interface. There was no desmoplastic stroma formation, and the tumour cells were separated from the hepatocytes by a thin layer of connective tissue fibres. A mild inflammatory infiltrate was nearly always present at the interface. The tissue architecture of the liver was not conserved within the metastases.

In the ‘replacement’ growth pattern, tumour cells were replacing hepatocytes in the liver plates, at the interface or throughout the metastasis, conserving the tissue architecture of the liver, without inflammation or fibrosis. Tumour cells and hepatocytes had intimate cell–cell contact. Intra- and interobserver variability of this classification was limited, as has been shown before (Vermeulen *et al*, 2001).

Glandular differentiation was graded according to the system of Elston and Ellis for BC (Elston and Ellis, 1991). For CRC metastases, the same grading system was used.

Immunohistochemical staining for CA9 was performed at the Weatherall Institute of Molecular Medicine (John Radcliffe Hospital, Headington, Oxford, UK) with the murine monoclonal antibody M75 at a dilution of 1 : 50, as has been described before (Chia *et al*, 2001). Bile ducts were used as an internal positive control, given the constitutive hypoxia-independent expression of CA9 by bile duct epithelium. The immunostaining was quantified by semiquantitative scoring: a score of 0–3 for the intensity of staining in the majority of the tumour cells was given (0, no staining; 1, weak staining; 2, moderate staining; 3, strong staining). The fraction (%) of immunostained tumour cells was estimated. The product of intensity score and percentage yielded a final global CA9 score of 0–300. The interface (about 20 tumour cell rows adjacent to the liver parenchyma) and the centre of the metastases were also evaluated separately for CA9 expression: any percentage of immunostaining in the respective regions was scored as positive CA9 expression.

Macrophages were immunostained with an anti-CD68 monoclonal antibody (clone KP-1, dilution of 1 : 80, DakoCytomation, Glostrup, Denmark) on the Ventana NexES automated immunostainer. Quantification of the relative immunostained area, as a measure of the number of macrophages, was performed with the Chalkley method, a point counting method using a microscope eyepiece graticule (Chalkley *et al*, 1943). The × 400 field at the tumour–liver parenchyma interface giving the impression of the highest number of CD68-positive cells on low magnification was selected and the Chalkley count was performed. From this field, an imaginary cross through the centre of the metastasis and with 90° angles was constructed, and the three other fields at the intersection of this cross and the interface were analysed. If a field contained a portal tract, the adjacent × 400 field was taken, given the high number of inflammatory cells present in portal tracts independent of the degree of inflammation at the interface elsewhere. The mean of the four counts was used for further analyses.

The presence of fibrin was detected immunohistochemically with the NYB.T2G1 monoclonal antibody (Accurate Chemical and Scientific Corp., Westbury, NY, USA; dilution 1 : 100), which reacts with the amino-terminal part of the Bß chain only after removal of fibrinopeptide B by thrombin, and hence binds to fibrin but not to fibrinogen. The staining was performed on the DAKO Autostainer (DakoCytomation) after pretreatment at 98°C for 30 min in Target Retrieval Solution (DakoCytomation). Fibrin deposition due to tissue damage during excision/biopsy of the metastases was usually present at the cut surface and was used as internal positive control. Negative staining in the absence of internal positive control staining resulted in exclusion of the result for further analysis. In all other cases, fibrin staining was graded from 0 to 3 (0: no staining; 1: minimal staining; 2: moderate staining; 3: extensive staining). This evaluation was performed both for the interface and the centre of the metastases.

Immunohistochemical staining for LYVE-1 was carried out as described previously (Beasley *et al*, 2002; Williams *et al*, 2003). In brief, antigen retrieval at 98°C for 30 min in Target Retrieval Solution (DakoCytomation) was followed by incubation with the affinity-purified LYVE-1 Ig (0.5 *μ*g ml^−1^) for 30 min. The staining was performed using the DAKO Autostainer (DakoCytomation peroxidase Envision kit).

Statistical analyses were performed using JMP-5 software (SAS Institute, North Carolina, USA) on an Apple PowerBook G4 (Apple Computer, California, USA).

## RESULTS

**Figure 1:**
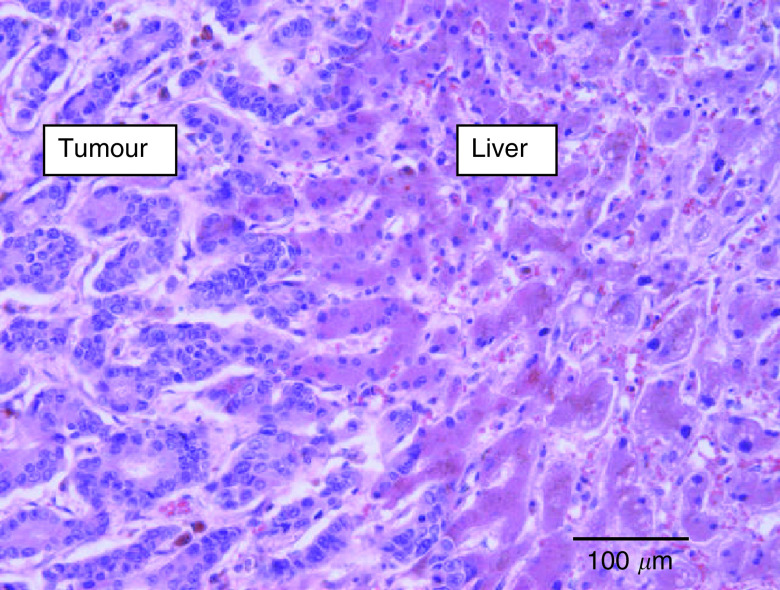
Breast cancer liver metastasis, replacement growth pattern (haematoxylin and eosin stain): the tissue architecture of the liver is preserved within the tumour tissue. There is close contact between tumour cells and hepatocytes at the interface and no inflammation.

In four BC metastases, the interface, and thus the growth pattern, could not be evaluated properly, and these cases were excluded from further analysis. The majority of the BC liver metastases showed a replacement growth pattern (96%) (Figure 1

; Table 1

**Table 1:** Distribution of the growth patterns of the 73 liver metastases according to the site of the primary tumour (*χ*^2^ analysis; Pearson test)

		**Desmoplastic**	**Pushing**	**Replacement**
Breast	(*n=*45)	1 (2%)	1 (2%)	43 (96%)
Colorectal	(*n=*28)	14 (50%)	5 (18%)	9 (32 %)
Total	(*n=*73)	15 (21%)	6 (8%)	52 (71%)
*P*<0.0001				

). In contrast, only one-third of the CRC liver metastases had these growth characteristics (32%), while 50% clearly induced a desmoplastic tissue reaction at the liver parenchyma–tumour interface (*χ*^2^ test *P*<0.0001) (Figure 2

**Figure 2:**
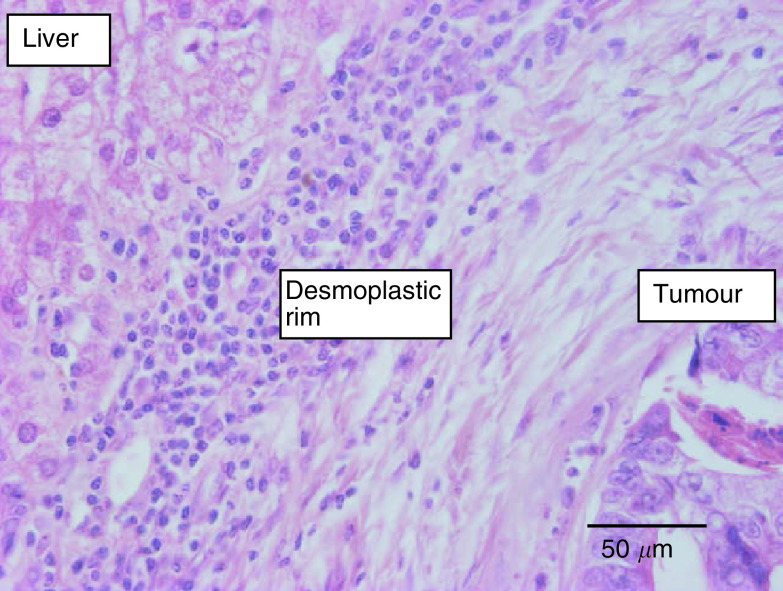
Colorectal cancer liver metastasis, desmoplastic growth pattern (haematoxylin and eosin stain): a rim of desmoplastic stroma separates the liver parenchyma from the tumour tissue. A dense inflammatory cell infiltrate is present in the stroma nearby the liver parenchyma.

). The characteristics of the replacement growth pattern were often present from interface up to the centre in the BC cases, while they were limited to the interface in all CRC liver metastases. In 15 metastases (eight of BC origin and seven of CRC origin), a mixed growth pattern was found. Only the dominant pattern was considered for further analysis.

When comparing BC liver metastases with CRC liver metastases (Table 2

**Table 2:** Comparison of glandular differentiation, fibrin deposition, CA9 expression and the macrophage content of breast cancer and colorectal cancer liver metastases

	**Breast**	**Colorectal**	
*Glandular differentiation*	(*n=*45)	(*n=*28)	
1	1 (3%)	21 (75%)	
2	8 (17%)	5 (18%)	
3	36 (80%)	2 (7%)	*P*<0.0001
			
*Fibrin, central*	(*n=*37)	(*n=*24)	
0	16 (43%)	6 (25%)	
1	10 (27%)	4 (17%)	
2	2 (6%)	4 (17%)	
3	9 (24%)	10 (41%)	*P=*0.15
			
*Fibrin, interface*	(*n=*38)	(*n=*25)	
0	30 (79%)	11 (44%)	
1	6 (15%)	10 (40%)	
2	1 (3%)	1 (4%)	
3	1 (3%)	3 (12%)	*P=*0.037
			
*CA9, central*	(*n=*44)	(*n=*24)	
−	32 (73%)	1 (4%)	
+	12 (27%)	23 (96%)	*P*<0.0001
			
*CA9, interface*	(*n=*45)	(*n=*24)	
−	38 (84%)	11 (46%)	
+	7 (16%)	13 (54%)	*P=*0.002
			
*Global CA9 score*	14.4±8.6 (0)	74.5±14.9 (52.5)	*P*<0.0001
			
*Macrophage count*	4.57±0.28 (4.25)	8.25±0.60 (7.50)	*P*<0.0001

**Figure 3:**
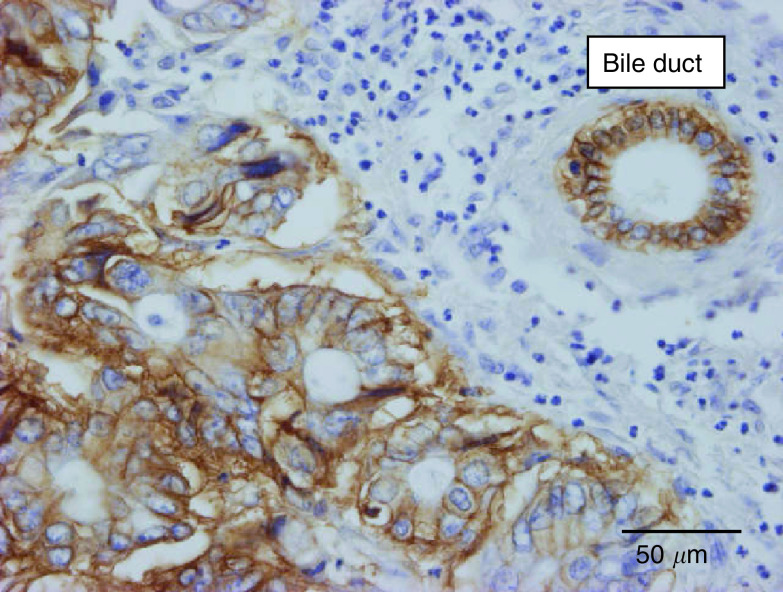
Carbonic anhydrase 9 immunostaining of a colorectal cancer liver metastasis: strong staining (brown; score 3+) of the tumour cells. Constitutive expression by bile duct epithelium (internal positive control).

Differences in categorical variables are validated by two-tailed Fisher's exact test. Continuous variables are expressed as mean±s.e. (median). Differences are validated by Wilcoxon test.

), the former showed less glandular differentiation (80% grade 3 *vs* 7% grade 3; *P*<0.0001), less frequently had fibrin deposition at the tumour–liver parenchyma interface (21 *vs* 56%; *P*=0.037), expressed CA9 only in a minority of cases at the interface (16 *vs* 54%; *P*=0.002) or in the central portion of the metastases (28 *vs* 96%; *P*<0.0001) (Figure 3

), and had a significantly lower macrophage Chalkley count (4.3 *vs* 7.5 (median); *P*<0.0001). After excluding the 28 necropsy-derived BC liver metastases, results were comparable (data not shown), with *P*-values of <0.0001 (glandular differentiation), 0.02 (central fibrin), 0.08 (fibrin at the interface), <0.0001 (central CA9), 0.009 (CA9 at the interface), 0.0004 (global CA9 score) and 0.001 (macrophage count).

Taking all liver metastases, there was a positive correlation between the global CA9 score and the Chalkley count of the macrophages (*r*=0.43; *P*=0.002).

**Figure 4:**
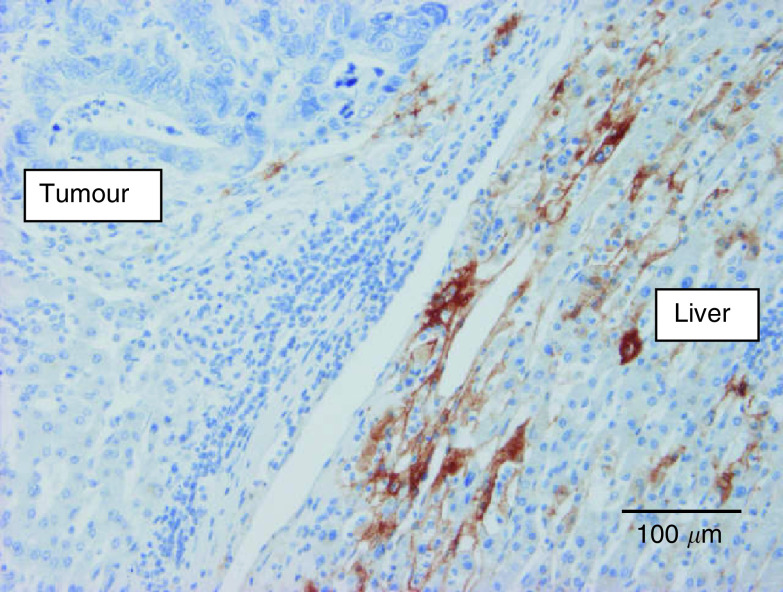
Immunostaining of fibrin (brown) in a desmoplastic colorectal cancer liver metastasis: fibrin deposits mainly in the liver parenchyma surrounding the metastasis.

**Figure 5:**
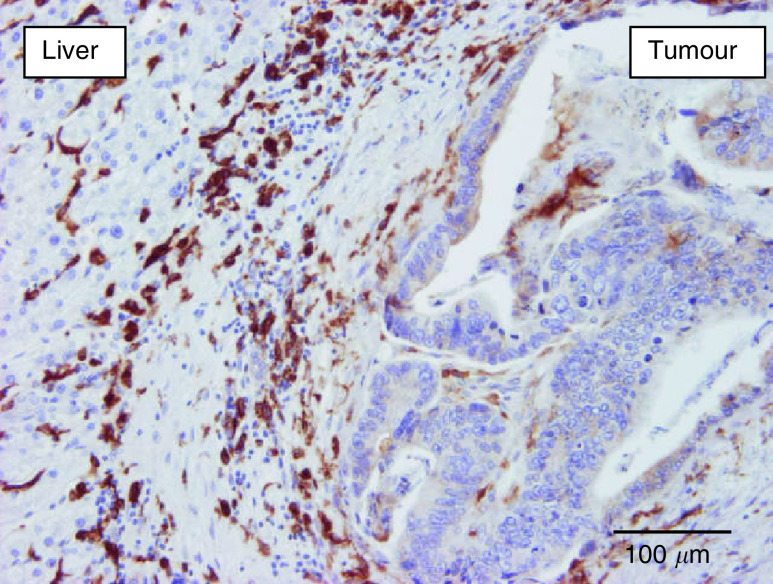
Anti-CD68 immunostaining demonstrating numerous macrophages (brown) in the desmoplastic rim surrounding a colorectal cancer liver metastasis. The Kupffer cells in the liver parenchyma are also immunoreactive.

Analysis of the influence of growth patterns on the different parameters was performed in the CRC liver metastases group and the BC liver metastases group separately. Forty per cent of the CRC liver metastases with a pushing growth pattern *vs* only 5% of the other liver metastases had extensive (grade 3) fibrin deposition at the interface (*P*=0.09) (Figure 4

). Desmoplastic CRC liver metastases had a mean macrophage Chalkley count of 9.3 (s.e.: 0.94; median: 9.5) *vs* 7.3 (s.e.: 0.69; median: 7.0) in the other CRC liver metastases (*P*=0.09) (Figure 5

). Although the majority of the non-replacement CRC liver metastases had CA9 expression at the tumour–liver parenchyma interface and only a minority of the replacement CRC liver metastases, this difference was not significant. Of the BC liver metastases with a replacement growth, only 24% had central CA9 expression, in contrast to all BC liver metastases with a non-replacement growth (*P*=0.06). Carbonic anhydrase 9 expression at the tumour–liver parenchyma interface was present in only 12% of the BC liver metastases with a replacement growth, in contrast to all BC liver metastases with a non-replacement growth (*P*=0.02). Other associations were not found.

**Figure 6:**
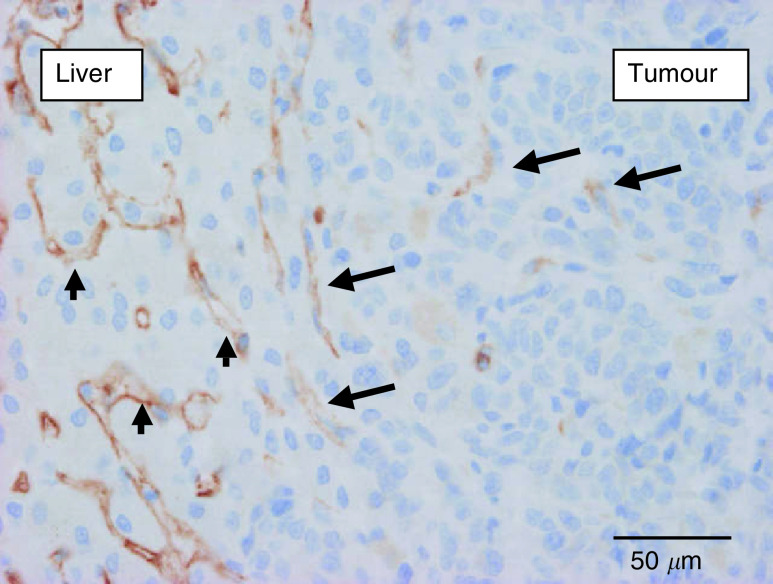
Expression of LYVE-1 (brown, immunostaining) by sinusoidal endothelial cells within the liver parenchyma at the interface with a replacement-type breast cancer liver metastasis (arrow heads). Sinusoids engulfed by tumour cells express LYVE-1 at the tumour–liver interface (arrows) and lose this expression towards the centre of the metastasis (towards the right on the microphotograph).

The lymphatic endothelial/sinusoidal marker LYVE-1 was expressed in the midzonal sinusoidal blood vessels of normal liver parenchyma, as was reported previously (Banerji *et al*, 1999). This constitutive expression was used as internal positive control of the immunohistochemical staining. In the desmoplastic CRC liver metastases, there were few if any LYVE-1-positive vessels in the connective tissue capsule or in the metastases. In most of the metastases with this growth pattern, LYVE-1 expression was absent or attenuated in the midzonal sinusoids of the surrounding liver parenchyma. In contrast, in the BC and CRC liver metastases with a replacement growth pattern, LYVE-1 expression in the sinusoids of the liver parenchyma that made contact with the tumour cells was not attenuated. Moreover, sinusoidal blood vessels engulfed by up to about 20 tumour cell rows still expressed LYVE-1 at the tumour–liver interface (Figure 6

), whereas those towards the centre of the metastases expressed less of the receptor.

## DISCUSSION

The liver is a target organ for metastasis of BC and CRC. The parenchyma is supported by a dense vasculature composed of branches from both venous and arterial blood vessels. Liver cell plates, usually composed of only two rows of hepatocytes, are intimately associated with sinusoidal blood vessels, in this way minimising the development of acute or chronic hypoxia in a tissue with a high cell density and a high metabolic activity. As hypoxia is the most important stimulus of angiogenesis, it was the hypothesis of this and a former study (Vermeulen *et al*, 2001) that liver metastases in which tumour cells would be able to preserve the architecture of the liver stroma could grow without hypoxia and subsequent angiogenesis. We have indeed shown that a minority of CRC liver metastases display a replacement pattern, characterised by low endothelial cell proliferation, a high tumour cell proliferation to endothelial cell proliferation ratio and only weak expression of CD34 in the constitutively CD34-negative endothelial cells of the co-opted sinusoidal blood vessels.

**Figure 7:**
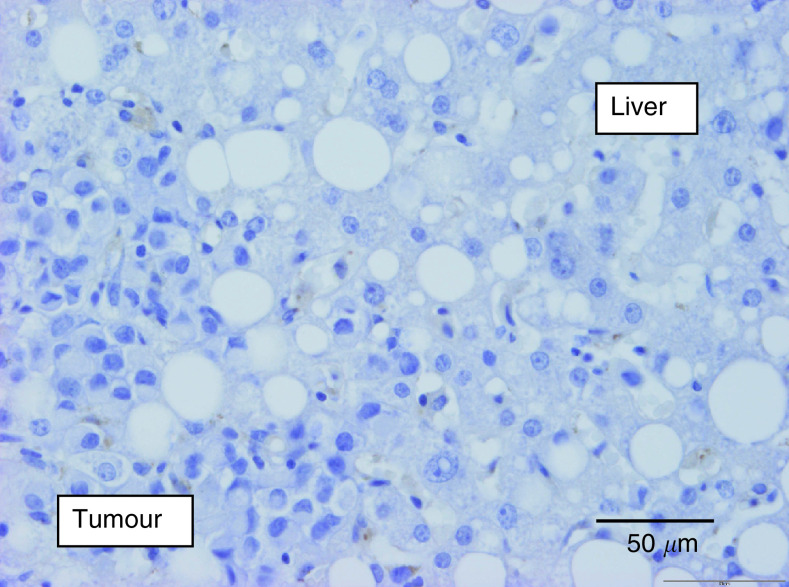
Carbonic anhydrase 9 immunostaining of a breast cancer liver metastasis: replacement growth and no immunostaining (constitutive expression by bile duct epithelium (internal positive control) was present in the section (not shown)).

In the present study, we have shown that BC liver metastases have different growth characteristics to CRC metastases: nearly all BC metastases had a replacement pattern that, in contrast to CRC metastases, was often also present in the centre of the metastases. In addition, this was characterised by minimal fibrin deposition, lack of CA9 expression in most of the BC metastases and a lower macrophage content compared to CRC metastases (Figures 7

, 8

**Figure 8:**
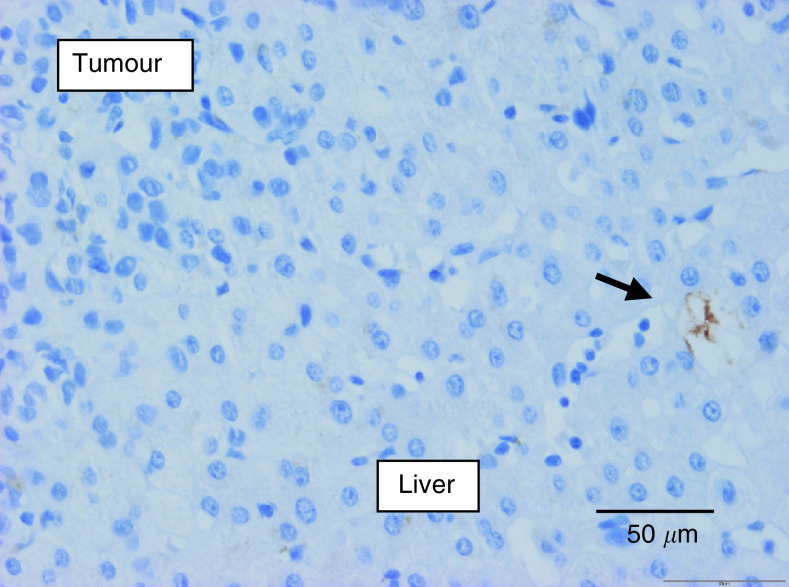
Immunostaining of fibrin (brown) in a replacement breast cancer liver metastasis: no staining at the tumour–liver interface (internal positive control: fibrin in a sinusoidal blood vessel (arrow)).

and 9

**Figure 9:**
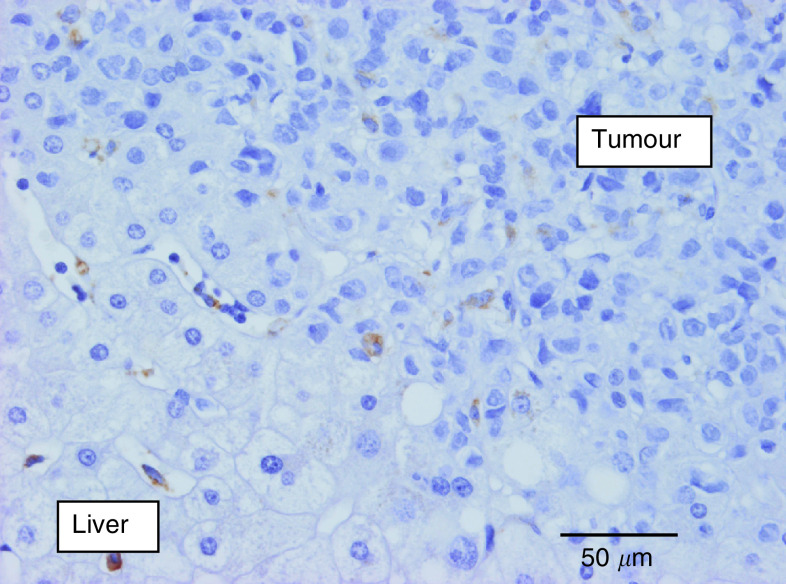
Anti-CD68 immunostaining demonstrating the Kupffer cells in the liver parenchyma. No macrophages at the tumour–liver interface of this breast cancer metastasis with replacement growth.

).

The induction of angiogenesis by breast cancer has been shown to be highly variable when comparing patients. In a study of cutaneous breast cancer deposits, an infiltrative growth pattern, in which cancer cells respect the dermal architecture and co-opt pre-existing blood vessels, was characterised by CA9 expression in only 17% of the tumours (Colpaert *et al*, 2003a). Fibrin was present in the dermal stroma in 33% of the deposits with an infiltrative growth pattern, and the endothelial cell proliferation was 4.2% (median value). For the expansive growth pattern, the respective values were 71, 89 and 16.4% (respective *P*-values: 0.02; 0.02; 0.004). Fifty-one per cent of the cutaneous deposits expressed the infiltrative, less or non-angiogenic growth pattern *vs* 18% with an expansive and angiogenic growth. The remainder of the metastases had a mixed growth pattern with intermediate characteristics. Also, primary BC has a variable angiogenic profile: inflammatory BC, a highly aggressive subtype with extensive dermal lymphovascular permeation, has been shown to have an endothelial cell proliferation fraction two-fold higher than non-inflammatory BC (19 *vs* 11%, respectively; *P*=0.01) (Colpaert *et al*, 2003b). Liver metastases of BC apparently are at the other end of the angiogenic spectrum: the majority of the BC liver metastases co-opt the pre-existing sinusoidal blood vessels in a replacement growth pattern that has been shown to have a low proliferative activity of the endothelial cells (Vermeulen *et al*, 2001).

**Figure 10:**
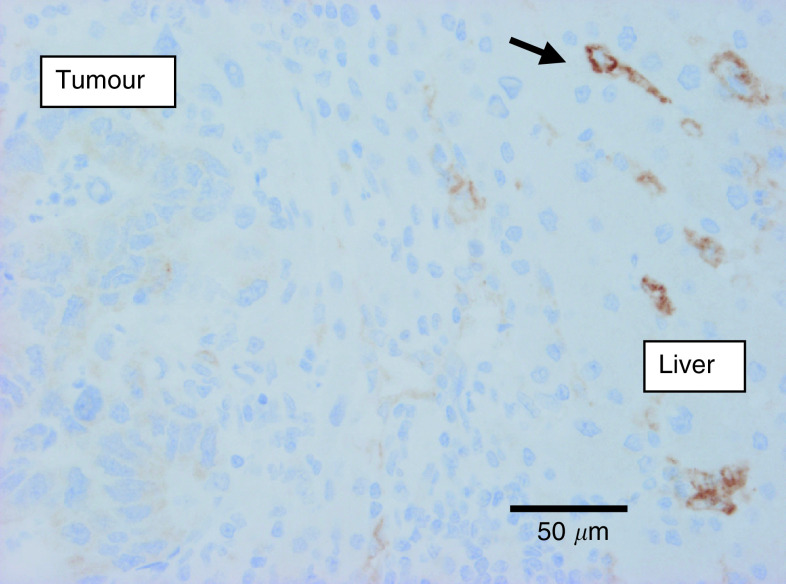
Expression of LYVE-1 (brown, immunostaining) by sinusoidal endothelial cells in the liver parenchyma surrounding a desmoplastic colorectal liver metastasis (arrows). No staining within the tumour tissue.

LYVE-1 is a receptor for hyaluronan mainly expressed on lymphatic endothelial cells (Banerji *et al*, 1999). Together with the liver sinusoids, the lymphatic system is responsible for the degradation of hyaluronan via LYVE-1. Expression of LYVE-1 has indeed been demonstrated on the endothelial cells of liver and spleen sinusoidal blood vessels (Banerji *et al*, 1999). In the liver metastases of BC and CRC with a replacement growth pattern, the blood vessels close to the interface, but well surrounded by tumour cells, continued to express LYVE-1. This observation, together with the conserved stromal architecture within the replacement-type metastases, strengthens the blood vessel co-option hypothesis. Interestingly, the co-opted sinusoidal blood vessels in the replacement growth pattern started to express CD34, which is not expressed constitutively on sinusoidal endothelial cells, at a distance of a few cell layers from the interface (Vermeulen *et al*, 2001). Both the apparent loss of LYVE-1 expression and the gain of CD34 expression indicate paracrine interactions between tumour cells and co-opted endothelial cells, yet without eliciting angiogenesis or desmoplasia. Recently, Williams *et al* (2003) have also reported the apparent loss of LYVE-1 from lymph vessels that were engulfed by invasive breast cancer cells. The possible mechanisms of this loss and its physiological consequences are currently under investigation (DG Jackson, unpublished). The desmoplastic-type metastases contained very few, if any, LYVE-1-expressing vessels, supporting the other data of angiogenesis as means of vascularisation in this growth pattern (Figures 10

).

A possible explanation for the higher fraction of angiogenic, desmoplastic liver metastases of CRC compared to BC might be that primary CRC are more angiogenic than primary BC. Indeed, using the same method for both tumour histiotypes to quantify angiogenesis by counting proliferating endothelial cells, the median proliferation fraction was more than six times higher in primary CRC than in BC (8.9 *vs* 1.4%, respectively; *P*<0.0001) (Vermeulen *et al*, 1997). This supports the rationale of counting microvessels in vascular ‘hot spots’ as a prognostic marker in solid human tumours: stroma containing more newly formed blood vessels is more likely to give rise to angiogenic metastases. Treatment of patients with metastatic colorectal cancer with bevacizumab, an anti-VEGF monoclonal antibody, has shown a clear synergy with chemotherapy, supporting the high angiogenic activity measured in our studies of primary colorectal cancer and its metastases in the liver (Kabbinavar *et al*, 2003).

The angiogenic proteome of the metastases seems to be similar to that of the primary tumours, since even in the less-vascularised skin, about half of the BC metastases grow with minimal induction of vessel sprouting, reflected by low endothelial cell proliferation. Taken together, the ‘seed’ might have more influence on the growth pattern than the ‘soil’. Nevertheless, the more angiogenic CRC can induce liver metastases with a blood vessel co-opting replacement growth pattern, as shown by us (this study; Vermeulen *et al*, 2001) and by Prall *et al* (2003). Three types of invasion of the liver parenchyma have been described by the latter authors, based on the number of apoptotic hepatocytes at the tumour–liver interface and on the degree of compression of the reticular connective tissue of the liver parenchyma surrounding the metastases. A pattern that resembles the replacement pattern of our study was characterised by prominent apoptosis of hepatocytes at the interface, with conservation of the reticular connective tissue architecture of the liver, with debris, but without fibrosis. The other two invasion patterns showed decreasing destruction of hepatocytes and increasing compression of the stroma surrounding the metastases with the formation of a pseudocapsule. These invasion patterns correspond to our pushing and desmoplastic growth patterns.

Other interesting observations concerning growth patterns of liver metastases originate from animal tumour models. Solaun *et al* (2002) have described two subtypes of colon carcinoma liver metastases that differ regarding their position in the liver tissue and regarding their connection to the local microvasculature: the portal type and the sinusoidal type. In the sinusoidal type of metastases, the blood vessels were recruited, without disturbing the liver architecture. The portal type induced a desmoplastic stromal reaction that surrounded the metastases and disturbed the liver architecture. Necrotic areas frequently developed in these metastases. The histological microphotographs in this publication show similar growth patterns as the replacement-type and desmoplastic-type metastases of our study. The main difference, however, is that the two types of metastases develop in the same animal after injection of a single tumour cell line, while patients in our study have liver metastases with a single growth pattern, independent of their position in relation to a portal tract. Whether the source of the blood vessels of human liver metastases is associated with the growth pattern or with the route of entrance of tumour emboli, arterial or venous, is not clear.

In a second animal tumour model (Griffini *et al*, 1997), 27% of the colon carcinoma liver metastases were small and totally encapsulated by stroma, which was always connected with adjacent portal tracts. All these metastases grew distant from the liver surface and consisted of well-differentiated acini. The majority of the metastases, however, were larger in diameter, and were not surrounded by a capsule. The metastases consisted mainly of undifferentiated cancer cells in direct contact with hepatocytes and without much desmoplastic stromal reaction. Most of these metastases were in contact with the liver capsule. In our study, human BC liver metastases were poorly differentiated and in most of these metastases, there was no desmoplastic reaction. The low degree of differentiation in the replacement growth patterns might be the consequence of less reciprocal interactions between cells from the desmoplastic stroma and epithelial tumour cells. On the other hand, less-differentiated tumour cells might more easily adopt a hepatocyte-like phenotype, induced by the sinusoidal blood vessels, a process analogous to liver development during foetal life (Zaret, 2002).

If the replacement growth pattern is indeed composed of less-differentiated tumour tissue, a worse prognosis would be expected. We have investigated an analogous phenomenon in primary lung cancer (Sardari Nia *et al*, 2003), and found that the alveolar growth pattern according to Pezzella *et al* (1997) in lung tumours indeed predicts shorter disease-free and overall survival. These tumours co-opt the blood vessels of the alveolar septa and are comparable to the liver metastases with a replacement growth pattern. Computed tomographic (CT) imaging of liver metastases has been shown to be predictive for recurrence after hepatic resection (Yamaguchi *et al*, 2002). CT contrast-enhanced images of liver metastases were subtyped according to the shape of the metastases and the irregularity of the outline of the nodules. Liver metastases with the most irregular shape and contour were predictive of reduced 5-year disease-free survival. Although this implies that growth and vascularisation patterns might influence prognosis, it is not clear how the images relate to the histological findings. We are currently performing studies to elucidate this important question.

Liver metastases of CRC had a significantly higher number of macrophages at the interface with the liver than BC metastases. Inflammation and cancer progression are intimately linked (Coussens and Werb, 2002) and, for instance, in primary BC, the degree of vascularity was positively associated with the number of hot spots of macrophages expressing HIF-2alpha (Leek *et al*, 2002). Since the HIF-2alpha expression in macrophages is induced by hypoxia (Burke *et al*, 2002), and since macrophages in tumours migrate to hypoxic areas, their presence in CRC liver metastases indicates a lower oxygen tension, which accords with the elevated CA9 expression compared to BC liver metastases.

Both CA9 and VEGF-A are regulated by transcriptional HIF complexes (Wykoff *et al*, 2000). VEGF induces blood vessel hyperpermeability resulting in extravasation of fibrinogen. Fibrin is then formed upon contact with the subendothelial matrix, supporting angiogenesis and inducing a wound-healing response with tumour stroma generation (Dvorak, 1986). The angiogenic CRC liver metastasis indeed frequently contained fibrin in contrast to the non-angiogenic BC metastases.

Finally, since 28 of 49 BC metastases were necropsy-derived compared to none of the CRC liver metastases, which were usually resected together with the primary colorectal tumour, it might be that advanced stage and multiple chemotherapy courses have influenced the growth pattern of BC metastases. Exclusion of the necropsy-derived BC liver metastases and statistical re-analysis did however not change the results.

In conclusion, this study shows that liver metastases of BC mainly co-opt sinusoidal blood vessel during their growth, in contrast to most of the CRC metastases, that expand with concomitant hypoxia-driven angiogenesis. The vessel-co-opting replacement growth pattern and the angiogenic desmoplastic growth pattern have also been observed in animal tumour models. This histopathological study opens perspectives for the study of the mechanisms responsible for the differences in tumour vascularisation of liver metastases. Whether metastases with a replacement growth pattern can sustain a more intense hypoxic stress before inducing angiogenesis, or whether hypoxia is not an issue due to the co-option of highly functional sinusoidal blood vessels, is not clear. Another possible mechanism is that angiogenesis is suppressed by endogenous angiogenesis inhibitors, which overrule local VEGF production. Additionally, some angiogenesis inhibitors seem to be able to inhibit HIF-1-induced transcriptional activation of VEGF expression (Mabjeesh *et al*, 2003).

The clinical relevance of our study is corroborated by the observation in one of the animal models that endostatin, an endogenous angiogenesis inhibitor, inhibits the growth of liver metastases with an efficacy that varies according to the growth pattern (Solaun *et al*, 2002).
